# Urinalysis and Clinical Correlations in Patients with* P. vivax* or* P. falciparum* Malaria from Colombia

**DOI:** 10.1155/2017/7868535

**Published:** 2017-05-24

**Authors:** Alberto Tobón-Castaño, Sebastián Barrera Escobar, Cecilia Giraldo Castro

**Affiliations:** ^1^Malaria Group, Faculty of Medicine, University of Antioquia, Calle 70, No. 52-21, Medellin, Colombia; ^2^Faculty of Medicine, University of Antioquia, Medellin, Colombia

## Abstract

**Background:**

Urinalysis is a poorly reviewed diagnostic tool in malaria patients; its application can show the presence of severe malaria.

**Methods:**

Urinalysis was performed in a total of 620 patients diagnosed with malaria by thick blood smear; complications were classified according to WHO major criteria for severity and minor criteria according to the Colombian malaria guideline.

**Results:**

Severe or moderate clinical complications were diagnosed in 31.1% of patients, hepatic dysfunctions were diagnosed in 25.8%, anemia was diagnosed in 9.8%, thrombocytopenia was diagnosed in 7.7%, renal dysfunction was diagnosed in 4.8%, neurological and pulmonary complications were diagnosed in 2.1% and 2.4%, hypoglycemia was diagnosed in 1.1% of patients with blood glucose analysis, and acidosis was diagnosed in 10 of 25. Bilirubinuria was found in 24.3%, associated with urobilinuria, proteinuria, and increased specific gravity; urobilinuria was found in 30.6% associated with elevated serum bilirubin and alanine aminotransferase; 39.2% had proteinuria, associated with higher blood urea nitrogen, serum bilirubin, aspartate, alanine-transaminase, hematuria, and increased specific gravity. Severe or moderate liver and renal complications were associated with proteinuria and bilirubinuria. Urobilinuria was associated with thrombocytopenia and neurological and hepatic dysfunction. Ketonuria was associated with neurological dysfunctions.

**Conclusions:**

The most frequent alterations in the urinalysis were bilirubinuria, proteinuria, urobilinuria, and increased specific gravity, related to thrombocytopenia and liver, kidney, and neurological alterations.

## 1. Background

Malaria has long been recognized as a disease that primarily affects tropical countries worldwide, making it a major international public health problem. According to the 2015 WHO World Malaria report, the annual incidence of the disease was estimated at 214 million clinical cases with an estimated mortality of 438,000 cases, 90% of them in African people [1] and mostly among pregnant women and children under the age of 5. It is estimated that in 2014 malaria was the cause of 7% of global child mortality [2]. Although the risk of acquiring malaria in Latin America is low [3] in Colombia it is a serious public health problem. An estimated 25 million people are at risk in 85% of rural regions, located below 1600 meters above sea level, and have climatic and epidemiological conditions suitable for disease transmission [4]. In Colombia the most common etiologic agents are* P. vivax* (66%) and* P. falciparum* (34%) [1].

Traditionally serious cases of malaria occur in* P. falciparum* infections, but recently complications caused by* P. vivax* have become more prevalent [5]. Early recognition of clinical symptoms predictive of the onset of complications would allow prompt treatment and supportive care to prevent progression to death [6].

As the malaria infection progresses some clinical signs indicate the establishment of serious illness and are danger signs for a complicated phase of the disease [6]; the most frequently observed are jaundice, cough, dyspnea, tachypnea, cyanosis, persistent vomiting, neurological disorders, and dark urine [7–9]. Early detection of urine alterations could allow for screening of early liver or kidney damage [7]. Dark urine has been associated with hemoglobinuria [10], myoglobinuria [10], hematuria [7], and bilirubinuria [7, 11], all indicators of liver or kidney dysfunction [7, 12]. Other alterations in the urine of malaria patients have been poorly explored as signs of danger.

Studies on urinalysis abnormalities in malaria patients are limited. A study of urinalysis abnormalities in 600 patients in Sudan reported albuminuria (84.9%), cylinders (71.4%), pyuria (53.8%), and hematuria (45%) in patients with a positive thick smear [13]. Bilirubin and urobilinogen concentrations in urine were increased in samples from Nigerian patients with malaria, compared to uninfected patients (*P* < 0.05) [11]. Other publications described proteinuria [7, 14, 15], hematuria [14], and myoglobinuria in malaria patients [10].

Liver dysfunction, renal dysfunction, and severe anemia were reported as the most common complications in hospitalized Colombian patients in association with dark urine and jaundice [6, 7]. In malaria patients from Colombia, hemoglobinuria (86%), urobilinuria (66%), proteinuria (54%), hematuria (43%), and bilirubinuria (39%) were associated with dark urine; renal dysfunction was identified in these patients [7].

Dark urine in adult malaria patients has been linked to myoglobinuria and rhabdomyolysis [10, 16, 17]. Although rhabdomyolysis has been reported in pediatric and adult patients with* P. falciparum* malaria [16], it is not a widely reported event in the literature; observations in Gambian children show a relationship between rhabdomyolysis and disease severity [18]. The pathogenic mechanism by which rhabdomyolysis develops is still not fully understood; the sequestration of parasitized erythrocytes in the microvasculature of skeletal muscle has been postulated as a cause of myocyte damage and the subsequent myoglobin release [16].

Hemoglobinuria, hematuria, and bilirubinuria have not been associated with liver or kidney dysfunction in previous observations in Colombia [7]. Choluria, the product of increased conjugated bilirubin (DB) has been described in malaria patients developing liver dysfunction as well as in kidney dysfunction by the toxic action of bilirubin on renal epithelium [7, 19, 20]. Bilirubin appears in urine before other signs of liver dysfunction are apparent [21]; therefore it can be considered to be an early sign of liver damage [7].

This study aims to analyze the urine alterations in relation to the clinical status of malaria patients from low malaria endemic regions in Colombia and explore its usefulness to recognize organ or systemic injury.

## 2. Methods and Materials

### 2.1. Design and Study Population

An analysis was performed on clinical and laboratory records of 620 patients with malaria obtained in two previous analytical studies [8, 9] and from inpatient medical records from the city of Medellin. The reference population consisted of patients of all age groups (from 1 to 85 years old) with symptoms suggestive of malaria and a parasitological diagnosis confirmed by thick blood smear in first- or second-level hospitals and healthcare facilities in the municipalities of Turbo and Necoclí (Urabá, Antioquia), El Bagre (Bajo Cauca, Antioquia), Tumaco, Guapi, and Timbiquí (Pacific Coast). The patients enrolled were in clinical and epidemiological studies conducted by the Malaria Group between 1997 and 2007 and were patients referred from different regions to third-level hospitals in Medellin (Pablo Tobón Uribe and San Vicente de Paúl Hospitals) between 2005 and 2010. Convenience sampling was applied.

### 2.2. Clinical and Laboratorial Criteria

A urine sample was obtained following the instructions for processing Bayer's Multistix strips. The urine specimen was examined within 1 hour of collection or was refrigerated (2–8°C) and examined within 4 hours of collection. Urinalysis was made using criteria of abnormality:

1. Glucose: any positive reading
2. Bilirubin: any positive reading
3. Ketones: above 5 mg/dL
4. Blood: whole or hemolyzed erythrocytes, including traces
5. pH: greater than 7.4 (basic) or lower than 4.8 (acid)
6. Protein: ≥30 mg/dL
7. Urobilinogen: ≥2 mg/dL
8. Nitrites: any positive reading
9. Leukocytes: ≥70/*μ*L
10. Specific gravity: >1.020

The presence of traces was not considered as an abnormal test result, except for hematuria. Dark yellow, orange red, or brown color in the urine was considered as “dark urine.”

Complications were classified according to WHO major criteria [22] for severity and the proposed minor criteria for Colombia [23] as follows:

1. Liver dysfunction: severe: total bilirubin [TB] > 3 mg/dL and/or transaminases > 120 IU/L; mild: TB > 1.5–3.0 mg/dL and/or transaminases > 80–120 IU/L.
2. Thrombocytopenia: profound: <25,000 platelets/*μ*L; severe: 25–50,000 platelets/*μ*L.
3. Renal dysfunction: severe: blood urea nitrogen [BUN] > 60 mg/dL and/or creatinine > 3 mg/dL; mild: BUN 41–60 mg/dL and/or creatinine 1.5–3.0 mg/dL.
4. Anemia: severe: hemoglobin [Hb] < 5.0 g/dL; moderate: Hb 5.0–6.9 g/dL.
5. Neurologic complication: cerebral malaria: seizures/coma; extreme weakness.
6. Pulmonary injury: acute respiratory distress syndrome (ARDS), pulmonary edema, or pleural effusion
7. Acid-base disturbances: severe acidosis: pH < 7.35 and HCO3 < 15 mEq/L; acidosis: pH < 7.35 and HCO3 15–18 mEq/L.
8. Hypoglycemia: severe: glycemia < 40 mg/dL; moderate: glycemia 40–49 mg/dL.

The patient must have at least one clinical criterion to be categorized as complicated.

### 2.3. Statistical Analysis

The Kolmogorov-Smirnov test was used to assess normality for quantitative variables; the median values were compared with the Mann-Whitney *U* test when variables were not normal. Associations between qualitative variables were analyzed with the Chi-square test; Odds Ratio (OR) and 95% confidence interval (CI) were estimated. Spearman's rank correlation coefficient was applied to analyze some relationships between numerical data. The adopted level of statistical significance was 5% (*P* < 0.05). The analysis was done with IBM® SPSS® statistics program, 22nd version (licensed to the University of Antioquia).

## 3. Results

**Figure 1:**
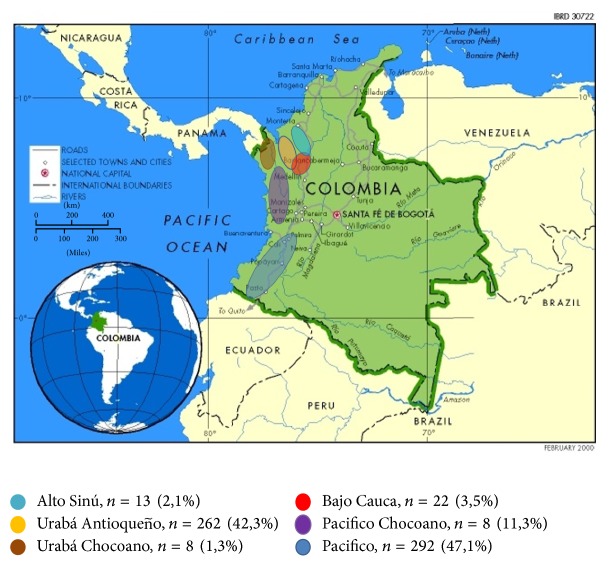
Geographical locations of the regions of origin of 618 patients, obtained and modified from the Cartographic Design Unit of the World Bank, 2000.

Urinalysis from 620 malaria patients was analyzed; some of the patients did not have all the urine dipstick parameters recorded. Patients had a median age of 22.8 years (mean 26; CI 24.7–27.2); 59.7% (*n* = 370) were men. Patients came from Urabá (Antioquia and Chocó) (43.6%), Pacific (50.5%), Bajo Cauca (3.5%), Alto Sinú (2.1%), and Magdalena Medio (0.3%) regions (Figure 1).* P. falciparum* infection was diagnosed in 419 (67.9%),* P. vivax* in 187 (30.2%), and mixed infection by these species in 14 (2.3%). Parasitaemia was distributed in 8 intervals; 68.6% had <10,000 parasites/*μ*L. During the course of the disease medication intake was reported as follows: 246 patients (39.7%) used analgesic or anti-inflammatory drugs, 31 (5.0%) antibiotics, 50 (8.0%) antimalarials, and 41 (7.0%) various medications (Table 1).

Severe or moderate clinical complications were diagnosed in 193 patients, 90 (14.5%) with severe complications and 103 (16.6%) with moderate complications, without statistical differences by species of plasmodium: 129 (66.8%) with* P. falciparum* infection, 54 (28%) with* P. vivax*, and 10 (5.2%) with mixed infections by these species (*P* > 0.05; Chi2). The complications include hepatic dysfunction in 160 (38.7%), anemia in 61 (9.8%), thrombocytopenia in 48 (7.7%), renal dysfunction in 30 (4.8%), and neurological and pulmonary complication in 13 (2.1%) and 15 (2.4%), respectively; hypoglycemia was diagnosed in 4 of 348 (1.1%) patients with blood glucose studies and acidosis in 10 of 25 (40%); no increased risk of alteration in the urinalysis was identified in hypoglycemic patients. No complicated malaria was diagnosed in 427 (68.9%) patients.

Urinalysis variables are described in Table 2; the relationships between urinalysis variables and hematological variables (Table 3) were analyzed.

### 3.1. Urine Color

The color was amber in 331 (79.2%) of the 418 analyzed samples, dark yellow in 64 (15.3%), orange in 11 (2.6%), red in 10 (2.4%), and brown in 2 (0.5%). Dark urine was statistically related to bilirubinuria (OR 6.7, CI 1.6–6.0, *P* < 0.001), blood (OR 3.6, CI 1.9–5.2, *P* < 0.001), hemoglobinuria (OR 3.1, CI 1.5–6.0, *P* = 0.001), hematuria (OR 2.2, CI 1.2–3.9, *P* = 0.006), urobilinogen (OR 2.8, CI 1.6–5.0, *P* < 0.001), and proteins (OR 5.8, CI 3.5–9.7, *P* < 0.001). Dark urine was related to severe or moderate hepatic complication (OR 3.1, CI 1.6–6.0, *P* = 0.001) but not renal dysfunction (*P* > 0.05). Serum BUN values > 20 mg/dL were more frequent in patients with dark urine (OR 2.2, CI 1.0–4.9, *P* = 0.053).

### 3.2. Nitrites and Leukocytes

Nitrites were found in 19 (3.4%) and leukocytes in 84 (14.9%) of 564 analyzed samples; these variables did not correlate with clinical complications.

### 3.3. pH

From 556 patients, 96.6% had a normal pH value (4.8 to 7.4) and 19 (3.4%) had a basic pH (≥7.4); these patients with a basic urinary pH had significantly lower serum values of BUN, TB, conjugated bilirubin [DB], and unconjugated bilirubin [IB] (Table 3) and a significant lower frequency of hepatic dysfunction (OR 0.6, CI 0.5–0.8) (Table 4).

### 3.4. Bilirubinuria

Present in 147 (24.3%) of 605 patients, bilirubinuria was associated with urobilinuria, proteinuria, and increased specific gravity (*P* < 0.05). Patients with bilirubinuria had significantly higher BUN, TB, DB, IB, and alanine aminotransferase [ALT] serum values (*P* < 0.05). The patients with complicated malaria and specifically with moderate or severe kidney or liver injury had a greater risk of bilirubinuria (Table 4). The hemoglobin values did not differ between patients with or without urinary bilirubin (*P* = 0.247, Mann-Whitney test).

### 3.5. Urobilinogen

Urobilinuria was identified in 184 (30.6%) of 602 patients; it was associated with higher bilirubin in urine and proteinuria (*P* < 0.05). Urobilinuria was related to increased serum BUN, BT, BD, BI, and ALT (*P* < 0.05) values; the median platelet count was lower in these patients than in those without urobilinuria (*P* < 0.001) (Table 3). The presence of urobilinogen was associated with an increased risk of severe malaria (OR 2.9, CI 1.9–4.5), specifically with moderate or severe complications: thrombocytopenia (OR 2.7, CI 1.3–5.3), neurological dysfunction (OR 1.1, IC 1.0–1.1), and hepatic dysfunction (OR 2.8, CI 1.7–4.5).

### 3.6. Ketones

Ketonuria (*n* = 48, 8.6%) was associated with proteinuria and increased specific gravity (*P* < 0.05). Ketonuria was not related to hematological alterations but it was associated with severe neurological dysfunction (OR 1.1, CI 1.0–1.2).

### 3.7. Glucose

Glycosuria was present in 14 of 564 (2.5%) patients; these patients had a higher risk of proteinuria (*P* < 0.05) and 9 of them had some criteria for severe malaria (OR 4.4; CI 1.4–13.2). The median blood glucose level was significantly higher in patients with glycosuria (*P* = 0.02).

### 3.8. Proteins

227 of 580 (39.2%) patients had proteinuria. This alteration was associated with bilirubinuria, urobilinuria, blood, and increased specific gravity (*P* < 0.05) and was related to higher serum BUN, TB, DB, ALT, and aspartate-transaminase (AST) values and lower platelet counts. Proteinuria was related to severe thrombocytopenia (OR 2.0, CI 1.1–3.8) and pulmonary (OR 4.0, CI 1.3–12.9) and liver complication (OR 1.5, CI 1.1–2.3); it was not statistically associated with renal complication but serum BUN values > 20 mg/dL were related to proteinuria (OR 2.9, CI 1.6–5.3, *P* < 0.001). Proteinuria was not related to severe anemia (*P* = 0.06) although patients with this complication had higher levels of proteinuria. In 25 complicated patients with lactic acid and bicarbonate measurements, acidosis was diagnosed in 9 and proteinuria was the most frequent abnormality (78%).

### 3.9. Blood in Urine

Blood was present in 182 (29.8%) of 602 analyzed samples, 82 (13.4%) classified as hemoglobinuria and 100 (16.4%) as hematuria. The presence of blood was associated with proteinuria (*P* < 0.05). There was no increased risk of clinical complications related to this alteration.

### 3.10. Specific Gravity

This variable was increased in 231 (41.2%) of 562 patients, who exhibited an increased risk of severe or moderate renal dysfunction (OR 1.7, CI 1.0–2.7, *P* = 0.033). The use of analgesics and anti-inflammatory was associated with higher specific gravity of urine (OR = 1.7; 1.9–2.6; *P* = 0.007).

Some alterations in the urine dipstick were related to other urinary alterations. Specific gravity was related to bilirubinuria (OR 1.7; CI 1.0–2.7; *P* 0.023), ketones (OR 2.3; CI 1.5–3.5; *P* 0.023), and proteins (OR 2.3; CI 1.5–3.5; *P* < 0.001). Blood in urine was related to proteins (OR 2.5; CI 1.7–3.6; *P* < 0.001). Bilirubinuria was related to proteins (OR 4.2; CI 2.8–6.4; *P* < 0.001) and urobilinogen (OR 4.7; CI 3.0–7.5; *P* < 0.001).

The malaria vivax patients exhibited a higher frequency of hematuria, hemoglobinuria, and leukocytes in urine than the* P. falciparum* cases, with significant statistical difference (*P* < 0.005, Chi-square test). In contrast, the* P. falciparum* cases showed higher frequency of ketonuria, proteinuria, and urobilinuria (*P* < 0.005, Chi-square test).

## 4. Discussion

The most common urinalysis alterations in the 620 malaria patients were increased specific gravity (41.2%), proteinuria (39.2%), urobilinuria (30.6%), blood (29.8%), bilirubinuria (24.3%), ketones (22.7%), and leukocytes (14.86%). Either one major or minor criterion of complication was diagnosed in* P. vivax* or* P. falciparum* cases without statistical differences, including thrombocytopenia, anemia, and liver and kidney dysfunction, consistent with other studies in Colombian patients which found liver and kidney complications and severe anemia [24].

The various parameters evaluated by urinalysis may be altered by a series of factors that reflect kidney or urinary tract alterations or systemic processes [12]. Proteinuria [7, 10, 13, 14], hematuria [7, 11, 13], bilirubinuria [7, 11, 13], hemoglobinuria [7, 13, 25, 26], and myoglobinuria [10, 15, 16] have been found in the urinalysis of patients with malaria.

### 4.1. Proteinuria

Proteinuria was found in 39.1% of samples, similar to previous findings in Colombian and Nigerian malaria patients [11, 15, 27]. In Colombian patients with* P. falciparum* infection, proteinuria was one of the most common alterations in urinalysis, reaching up to 54%, and was associated with renal dysfunction [7]. In our patients, proteinuria was associated with increased TB and DB serum levels, with increased risk of liver, kidney, and lung complications, and other alterations in urinalysis including bilirubinuria, urobilinuria, ketonuria, and glycosuria. Concurrent hematuria was found in 93 of 227 patients with proteinuria (40.96%) but hematuria was not related to clinical complications.

In malaria patients with acute kidney injury, proteinuria indicates progression to nephropathy [7, 18, 28, 29]. Proteinuria associated with blood in urine (hematuria or hemoglobinuria) suggests glomerular disease [15, 30] and a mixed pattern of glomerular-tubular excretion has been established in malaria patients [18, 31]. Observations in children with cerebral malaria in Gambia showed that 53% had a glomerular-tubular pattern of urinary protein excretion, while 46% had a tubular pattern [18], and postmortem histological evidence of glomerular lesions by* P. falciparum* has been described [20, 32–34]. Interstitial nephritis caused by influx of leukocytes to renal tissue could explain such alterations; however glomerulonephritis attributed to the deposition of immune complexes is a form of glomerular injury poorly reported in the malarial patient [19].

In our patients, there was a relationship between proteinuria and BUN (OR 2.8, *P* = 0.001) but not with creatinine. This finding is similar to observations by Hanson et al. [35] but contrasts with observations in India that found no relationship between proteinuria, serum creatinine, and BUN [14]. In 1328 Colombian patients, aged 5 to over 60 years, proteinuria was present in 54% of* P. falciparum* infections and 48% of* P. vivax*, and it was associated with serum creatinine > 1.5 mg/dL (*P* = 0.013) and with choluria and hemoglobinuria, suggesting kidney damage [15]. In 40 Nigerian children with acute falciparum malaria, the creatinine clearance rate [CCR] was significantly lower during the acute phase of infection compared to the recovery stage; proteinuria was presented in 40% of patients but was not associated with CCR [27].

Increased BUN or creatinine can result from prerenal azotemia [19] through an inadequate kidney blood flow and a glomerular hydrostatic pressure insufficient to maintain the glomerular filtration rate [GFR] [36]. Long lasting prerenal azotemia can produce tubular necrosis, the most common form of renal injury in malaria [19]. However the diagnosis of renal damage is traditionally supported on serum creatinine increases (values over 1 mg/dL) and these values are only apparent once the GFR has fallen about 50% [37]. Proteinuria may be an early result of injury before a significant drop in the GFR and creatinine rise can be observed. In our patients, relation between proteinuria and severe kidney injury was not found (creatinine > 1,5 mg/dL or BUN > 40 mg/dL; *n* = 30), but when severe and mild kidney injury (Cr > 1 mg/dL or BUN > 20 mg/dL; *n* = 170) were pooled, a relation was established between proteinuria and kidney injury (OR 1.7; CI 1.0–2.6; *P* = 0.029), possibly due to the greater number of subjects included in the analysis. In the same direction, proteinuria was related to BUN > 20 mg/dL (OR 2.9; CI 1.6–5.3; *P* = 0.029). In Indian patients, proteinuria was an independent factor for kidney injury with a 88.2% sensitivity and 62.7% specificity, compared with the respective values of 94,1% and 90,8% for hematuria, the latter related to raised BUN and creatinine (*P* < 0.01) [14].

### 4.2. Bilirubinuria

The clinical manifestations of malaria generally include paroxysmal episodes of fever and chills every 24–48 hours or 72 hours depending on the infecting species [38, 39], which coincide with synchronous rupture of parasitized erythrocytes [3]. Circulating bilirubin levels increase due to hemolysis during erythrocytic schizogony; this hyperbilirubinemia is mild and does not generate intense jaundice [40, 41]. Increased levels of conjugated and total bilirubin have been reported in malaria patients [7, 11, 41, 42], possibly due to an increased hepatic conjugation of unconjugated bilirubin [7]. Significant hyperbilirubinemia is an important marker of liver dysfunction with an increase in aminotransferases [7, 43]. Under normal conditions the bilirubin increase in blood is offset by the increase of bilirubin excretion in urine to prevent hyperbilirubinemia [44]. Conjugated bilirubin is water soluble and therefore may be detected in the urinalysis, while unconjugated bilirubin, insoluble, cannot pass through the renal glomerulus and cannot be detected in the test [12]. Both conjugated and unconjugated bilirubin are toxic to the kidney tissue [19, 45] especially to renal tubular cells, hence the fact that a patient with hyperbilirubinemia has a greater risk of tubular necrosis [7].

A higher conjugated bilirubin concentration in blood and increased mortality in patients with impaired renal function in falciparum malaria cases in India has been reported; renal function deterioration was associated with a decrease in excretion of conjugated bilirubin in urine [45]. A lower rate of bilirubin excretion in urine may lead to an increase in conjugated bilirubin concentration in blood, perpetuating a cycle of renal injury and predisposing these patients with renal injury to develop other complications [45] such as cerebral malaria, electrolyte disorders, jaundice, and anemia [19, 20, 32].

Moderate or severe liver complication was present in 38.7% of our patients, consistent with previous studies that found up to 33% of patients with this complication [9]. In these patients, both proteinuria (OR 1.5, CI 1.0–2.3) and bilirubinuria (OR 2.1, CI 1.3–3.3) were associated with an increased risk of liver complications; and both were related to renal complications (*P* < 0.05), suggesting a role as predictors of injury in these organs since kidney and liver dysfunction are involved in other clinical complications in malaria patients. From this evidence we could then explain the association between proteinuria and increased TB and DB by a complex mechanism that involves not only the direct toxic effect of bilirubin on tubular cells [7, 45], but also the effects of prerenal azotemia which in many cases can lead to the development of ischemic damage often described as acute tubular necrosis [36]. The mechanisms that lead to all of these alterations in urinalysis parameters share a nature beyond the local renal commitment and involve factors both within and outside this organ, evidenced by the complex pathogenesis of the establishment of acute kidney injury in the malarial patient [19].

### 4.3. Other Relations with Liver and Kidney Injury

Associations were found among serum ALT and bilirubinuria (*P* = 0.01), urobilinuria (*P* = 0.006), and proteinuria (*P* = 0.001); this contributes to explaining a relationship between liver complication and bilirubinuria (OR 2.1, CI 1.3–3.3), urobilinuria (OR 2.7, CI 1.7–4.5), and proteinuria (OR 1.5, CI 1.0–2.3).

Pyuria was detected in 22.9% of our sample, a parameter rarely reported in malaria. In Nigeria, pyuria was informed in malaria patients but without difference in the frequency with nonmalaria patients [11]. In Sudan, pyuria was found in 140 patients, 53.8% with a positive blood smear, but the difference between the occurrences of pyuria in malarial and nonmalarial patients was not statistically significant [13]. The presence of leukocytes in urine is highly specific to urinary tract infection but it has low sensitivity; combined with a positive nitrite test it is a strong indicator of infection. However, nitrites may be affected for many reasons including bacteria lacking the enzyme that converts nitrates to nitrites, so a negative result of the latter does not exclude the possibility of urinary tract infection [21]. A meta-analysis about bacterial infections in children infected with* P. falciparum* concluded that these patients were at an increased risk of bacterial infections resulting in increased mortality [46].

While in our study the presence of leukocytes was not related to an increased risk of clinical complications, it is worth noting this marker of urinary tract infection; this may reflect the different immunosuppressive events that occur during malaria. A greater susceptibility of the malaria patient to various infections during the acute disease has been described, along with the role of hemozoin as an immunosuppressant that affects both antigen processing and immunomodulatory effects in macrophages [47] as well as by inhibiting dendritic cell activity [48]; the inhibition of the neutrophil function has been related to the hemolysis and the heme oxygenase one induction [49]. The defense of the urinary tract relies primarily on the innate immunity, with a particular interest in the neutrophil function in the bladder [50]. In fact different polymorphism in the TLR (Toll-Like Receptor) genes has been related to an increased susceptibility to urinary tract infections [51]; as the malaria parasite can inhibit the innate immune function, increased urinary tract infections in the malaria patients could appear.

Finally, leukocyturia in patients could be related to interstitial nephritis; this along with hematuria can be present in different types of glomerulonephritis [52]; indeed the interstitial nephritis has been described in infections with* P. falciparum* in association with acute tubular necrosis and glomerular injury [53, 54].

This study found a relation between proteinuria and a high specific gravity (*P* < 0.05). Specific gravity in a urine sample depends on both the number of particles present in the sample and their weight [21]. Increased urine specific gravity may be due to increased solutes in the sample, such as proteins, bilirubin, and ketones. Impaired renal function must be considered as one of the factors that can increase specific gravity in urine [12].

Ketones was also associated with an increased risk of neurological complications (*P* = 0.002) but no relation with hematological variables was found. Ketones are a product of the decreased metabolism of glucose in the patients [12]; as glucose is one of the most important substrates for brain metabolism, the neurologic complications (manifested as seizure or coma) can appear. Although our patients with neurological complications have lower blood glucose levels than those without neurological alteration (median 90 versus 102 mg%), the difference is not significant. Additionally the low number of patients with neurological complication and blood glucose and urine ketones measurements (*n* = 5) prevents exploring these relationships.

In this study proteinuria has been related to moderate or severe pulmonary complication (*P* = 0.025); this complication has been reported in patients with malarial acute kidney injury [20, 33] and has been described in 31% of malarial patients who required treatment in an intensive care unit [55]. One possible explanation for this association can be the increased blood volume, by either renal or hydric overload; both conditions have been related to pulmonary complication in the malarial patients [56]. In fact the association between malaria kidney complication and pulmonary complication was evaluated in a study by Kaushik et al. in malaria vivax patients in India; they found that 14% of the patients with acute kidney injury also presented acute respiratory distress syndrome [57].

The relationship between analgesic and anti-inflammatories use and the increased urinary density is in favor of the association between this alteration and renal dysfunction. In addition to malaria, kidney injury may be favored by the use of medications. One interesting finding is the association between urobilinogen and moderate or severe thrombocytopenia; this can reflect the severity of the disease.

Dark urine was related to proteins, urobilinogen, bilirubin, and blood (hemoglobin or hematuria) in urine, associated with liver dysfunction, and was more frequent in patients with BUN over 20 mg/dL. Previous findings reported no association between dark urine and hepatic or renal complication, concluding that this sign can be used as early marker of these complications [8]. Bilirubin in urine is, almost entirely, conjugated bilirubin and evidences hepatic injury, in the same way of other findings in this study.

A limitation of the study is that a multivariate model of the relationships between urinalysis and severe malaria was not obtained, probably because this outcome is due to different complications; only bivariate relations were obtained. Since the pathogenesis underlying each organ injury would produce distinct alterations in the urine, an important statistical sample of each complication would be required.

## 5. Conclusions

The most frequent alterations in the urinalysis in Colombian patients with malaria were bilirubinuria (24.3%), urobilinuria (30.6%), proteinuria (39.2%), and high density (41.2%) related to liver, kidney, and neurological function and thrombocytopenia. The use of the urine dipstick facilitated rapid identification of liver and kidney injury and other suspected complications including acidosis, pulmonary, and neurologic compromise.

Urinalysis proves to be an excellent tool to detect pathological changes in malaria patients. Its low cost and ease of application at the bedside make it a cost effective method applicable in the first levels of care for the timely recognition of patients at risk allowing appropriate care to reduce severe morbidity and deaths. The relationship between alterations in urine and serum markers of organ dysfunction indicates that this test is useful to alert the clinician to the progression of malaria to severe disease.

## Figures and Tables

**Table 1:** Sociodemographic characteristics.

Variable (*n* = 620)	Severe malaria	Uncomplicated malaria	Total frequency	*P* value
Sex				**0.009**
Male	129 (34.9%)	241 (65.1%)	370 (59.7%)	
Female	64 (25.6%)	186 (74.4%)	250 (40.3%)	
Ethnicity^**∗**^ (race)				**<0.001**
Mestizo	125 (37.4%)	209 (62.6%)	334 (57.6%)	
Afro	21 (10.8%)	174 (89.2%)	195 (33.6%)	
White	14 (35.9%)	25 (64.1%)	39 (6.7%)	
Amerindian	2 (16.7%)	10 (83.3%)	12 (2.1%)	
Residence^**∗∗**^				0.188
Rural area	98 (42.2%)	134 (57.8%)	232 (61.9%)	
Urban	53 (37.1%)	90 (62.9%)	143 (38.1%)	
Age ranges				0.129
1–5 years	10 (40.0%)	15 (60.0%)	25 (4.0%)	
5.1–15 years	36 (23.3%)	112 (75.7%)	148 (23.9%)	
15.1–65 years	140 (32.5%)	291 (67.5%)	431 (69.5%)	
>65 years	7 (43.7%)	9 (56.3%)	16 (2.6%)	
Species				**0.004**
*P. vivax*	54 (28.9%)	133 (71.1%)	187 (30.2%)	
*P. falciparum*	129 (30.8%)	290 (69.2%)	419 (67.6%)	
Mixed	10 (71.4%)	4 (28.6%)	14 (2.2%)	
Parasitemia ranges^**∗∗∗**^				**<0.001**
<1,000	24 (22.6%)	82 (77.4%)	106 (18.0%)	
1,000–5,000	42 (24.9%)	127 (75.1%)	169 (28.7%)	
5,001–10,000	38 (29.5%)	91 (70.5%)	129 (21.9%)	
10,001–50,000	54 (34.4%)	103 (65.6%)	157 (26.7%)	
50,001–100,000	13 (61.9%)	8 (38.1%)	21 (3.6%)	
>100,000	6 (100%)		6 (1.0%)	
Previous use of any drug				0.052
Yes	18 (46.9%)	23 (56.1%)	41 (6.6%)	
Previous use of antimalarials				**<0.001**
Yes	31 (62%)	19 (38%)	50 (8.1%)	
Previous use of antibiotics				0.129
Yes	13 (41.9%)	18 (58.1%)	31 (5.0%)	
Use of analgesics and/or anti-inflammatory drugs				**<0.001**
Yes	101 (41.1%)	145 (58.9%)	246 (39.7%)	

^*∗*^ *n* = 580; ^*∗∗*^*n* = 375; ^*∗∗∗*^*n* = 588.

**Table 2:** Urinalysis test variables in 620 patients with malaria^*∗*^.

*Specific gravity* (*n* = 562)	*1,000*	*1,005*	*1,006*	*1,01*	*1,015*	*1,02*	*1,025*	*1,03*
	3 (0.5%)	24 (4.3%)	1 (0.2%)	62 (11%)	76 (13.5%)	166 (29.5%)	111 (19.8%)	120 (21.4%)

*pH* (*n* = 556)	*5.5*	*6.0*	*6.5*	*7.0*	*7.5*		*8.0*	*8.5*
	87 (15.6%)	374 (67.3%)	30 (5.4%)	46 (8.3)	3 (0.5)		15 (2.7%)	1 (0.2%)

*Blood* (*n* = 610)	*Negative*	*Traces*	*Moderate*	*Traces*	*1+*		*2+*	*3+*
		(not lysed)	(not lysed)	(lysed)	(lysed)		(lysed)	(lysed)
	428 (70.2%)	83 (13.6%)	17 (2.8%)	7 (1.1%)	15 (2.5%)		17 (2.8%)	43 (7%)

*Proteins* (mg/dL) (*n* = 580)	*0 Negative*	*Traces*	*30.0*		*100*		*300*	*≥2,000*
	202 (34.8%)	151 (26%)	152 (26.2%)		66 (11.4%)		7 (1.2%)	2 (0.3%)

*Urobilinogen* (mg/dL) (*n* = 602)	*0.2, negative*		*1.0 traces*		*2.0*		*4.0*	*≥8.0*
	418 (69.4%)		78 (13%)		17 (2.8%)		51 (8.5%)	38 (6.3%)

*Leukocytes* (cells/*μ*L) (*n* = 564)	*Negative*		*15 traces*		*70*		*125*	*500*
	480 (85.1%)		45 (8.0%)		28 (5.0%)		8 (1.4%)	3 (0.53%)

*Ketones* (mg/dL) (*n* = 559)	*0, negative*		*5.0 traces*		*15 (low)*		*40 (moderate)*	*≥80 (high)* ^*∗∗*^
	432 (77.3%)		79 (14.1%)		19 (3.4%)		15 (2.7%)	14 (2.5%)

*Glucose* (mg/dL) (*n* = 564)	*Negative (<30.0)*		*100*		*250*		*500*	*1*
	550 (97.5%)		6 (1.1%)		5 (0.9%)		18 (0.2%)	2 (0.4%)

*Bilirubin* (mg/dL) (*n* = 605)	*Negative (<0.2)*		*Low*		*Moderate*		*High*	
	458 (75.7%)		102 (16.8%)		36 (6%)		9 (1.5%)	

*Nitrites* (*n* = 564)	*Negative*				*Positive*			
	545 (96.6%)				19 (3.4%)			

^**∗**^Not all patients have complete urine dipstick variables; ^**∗∗**^only one patient with ketones > 160 mg/dL.

**Table 3:** Comparison of hematological values (median) based on the urinalysis findings in 620 patients with malaria.

	Blood^*∗*^ (no/yes)	Glucose (no/yes)	Bilirubin (no/yes)	Ketones (no/yes)	Elevated specific gravity (no/yes)	Basic pH (>7,4) (no/yes)	Urobilinogen (No/Yes)	Proteins (No/Yes)
Creatinine, mg/dL *P* value	(1.0/1.0) 0.467	(1.0/1.1) 0.46	(0.98/1.0) 0.173	(1.0/0.9) 0.068	(0.95/1.0) 0.098	(1.0/0.9) 0.168	(1.0/0.9) 0.825	(0.95/1.0) 0.29
BUN, mg/dL *P* value	(13.69/11.89) 0.483	(12.3/15.0) 0.13	(12/14.1) **0.019**	(12.7/11.5) 0.711	(12.0/14.1) **0.012**	(12.69/9.4) **0.018**	(12.0/14.24) **0.004**	(11.28/14.42) **0.000**
Total bilirubin, mg/dL *P* value	(1.0/1.0) 0.972	(1.0/1.2) 0.445	(0.9/1.35) **0.00**	(1.0/0.99) 0.979	(0.96/1.0) 0.813	(1.0/0.66) **0.009**	(0.9/1.55) **0.000**	(0.90/1.24) **0.004**
Conjugated bilirubin, mg/dL *P* value	(0.33/0.35) 0.663	(0.3/0.4) 0.059	(0.3/0.43) **0.002**	(0.33/0.36) 0.537	(0.32/0.39) 0.259	(0.36/0.20) **0.013**	(3.0/6.0) **0.000**	(0.3/0.43) **0.000**
Unconjugated bilirubin, mg/dL *P* value	(0.66/0.61) 0.602	(0.6/0.66) 0.797	(0.6/0.81) **0.000**	(0.63/0.69) 0.82	(0.63/0.65) 0.475	(0.66/0.39) **0.035**	(0.6/0.97) **0.000**	(0.60/0.68) 0.094
AST IU/L *P* value	(36.0/36.0) 0.765	(36.75/58.0) 0.06	(36.0/37.0) 0.261	(37.0/37.0) 0.832	(37.0/33.0) 0.13	(37.0/28.0) 0.23	(36.0/36.0) 0.268	(33.0/40.0) **0.028**
ALT IU/L *P* value	(32.5/24.0) 0.269	(31.5/52.0) 0.302	(28.0/55.0) **0.001**	(32.0/32.0) 0.797	(31.0/33.0) 0.68	(31.5/31.0) 0.505	(27.5/47.0) **0.006**	(28.0/50.0) **0.001**
Hemoglobin, g/dL *P* value	(11.4/11,8) 0.255	(11.5/12.5) 0.386	(11.5/12.1) 0.247	(11.7/10.9) 0.22	(11.5/12.4) 0.148	(11.6/10.8) 0.413	(11.6/11.8) 0.75	(11.7/11.4) 0.516
Platelets (platelets/*μ*L) *P* value	(117,000/108,000) 0.345	(111,000/91,500) 0.26	(113,000/110,000) 0.304	(112,000/1,050,000) 0.844	(117,000/102,500) 0.378	(109,500/131,500) 0.242	(126,500/89,000) **0.000**	(131,000/96,000) **0.000**

^*∗*^Hematuria and/or hemoglobinuria. Analysis for hemoglobinuria indicates similar findings.

**Table 4:** Urinalysis variables according to the clinical complications.

Severe or moderate complication	pH		Bilirubin		Urobilinogen		Ketones		Proteins	
	Basic	Normal	Positive	Negative	Positive	Negative	Positive	Negative	Positive	Negative
Complicated malaria^**∗∗**^										
Yes	3	167	55	129	54	127	17	150	86	93
No	16	370	92	329	52	369	31	361	141	260
OR–CI	0.4	0.1–1.4	**1.5**	**1.0–2.3**	**3.0**	**2.0–4.5**	1.4	0.7–2.5	**1.7**	**1.2–2.4**
*P* value	0.167^*∗*^		**0.035**		**0.000**		0.381		**0.003**	
Thrombocytopenia										
Yes	1	42	14	30	15	25	1	41	25	18
No	13	283	76	265	63	278	35	263	130	188
OR–CI	0.5	0.1–4.1	1.6	0.8–3.2	**2.6**	**1.3–5.3**	0.2	0.1–1.2	**2.0**	**1.1–3.8**
*P* value	0.525^*∗*^		0.163		**0.006**		0.114^*∗*^		**0.034**	
Anemia										
Yes	3	52	7	51	8	46	5	49	30	26
No	11	300	90	264	79	276	32	281	133	199
OR–CI	1.6	0.4–5.8	**0.4**	**0.2–0.9**	0.6	0.3–1.3	0.9	0.3–2.4	1.7	1.0–3.0
*P* value	0.451^*∗*^		**0.031**		0.217		0.828		0.06	
Neurological complication										
Yes	0	11	1	11	6	4	4	6	6	5
No	19	526	146	447	100	491	44	505	221	348
OR – CI	NA	NA	0.3	0.1–2.2	**7.3** ^*∗*^	**2.0–26.5**	**7.6**	**2.1–28.1**	1.9	0.6–6.3
*P* value	NA		0.336^*∗*^		**0.001** ^*∗*^		**0.002** ^*∗*^		0.456	
Pulmonary complication										
Yes	0	13	3	10	4	6	2	11	10	4
No	19	524	144	448	102	490	46	500	217	349
OR–CI	NA	NA	0.9^*∗*^	0.3–3.4	3.2	0.9–11.5	1.9^*∗*^	0.4–9.2	**4.0** ^*∗*^	**1.3–12.9**
*P* value	NA		0.823^*∗*^		0.145^*∗*^		0.700^*∗*^		**0.025** ^*∗*^	
Liver complication										
Yes	1	140	50	101	48	102	14	124	72	77
No	11	202	48	199	36	210	21	194	85	140
OR–CI	**0.6**	**0.5–0.8**	**2.1**	**1.3–3.3**	**2.7**	**1.7–4.5**	1.0	0.5–2.1	**1.5**	**1.01–2.3**
*P* value	**0.032** ^*∗*^		**0.002**		**0.000**		0.908		**0.044**	
Renal complication										
Yes	0	26	5	20	8	15	1	25	14	13
No	14	316	92	283	75	299	33	296	143	206
OR–IC	ND	ND	0.8	0.2–2.1	2.1	0.9–5.2	0.4	0.1–2.7	1.5	0.7–3.4
*P* value	ND		0.787		0.098		0.323		0.273	

^**∗**^Fisher exact test; ^**∗∗**^any criteria of complication; ND: no data.

## Abbreviations

ALT: Alanine aminotransferase
AST: Aspartate aminotransferase
BUN: Blood urea nitrogen
Cr: Creatinine
CCR: Creatinine clearance rate
DB: Conjugated bilirubin
GFR: Glomerular filtration rate
Hb: Hemoglobin
TB: Total bilirubin
IB: Unconjugated bilirubin.
